# Comparative Analysis of ESBL-Positive *Escherichia coli* Isolates from Animals and Humans from the UK, The Netherlands and Germany

**DOI:** 10.1371/journal.pone.0075392

**Published:** 2013-09-26

**Authors:** Guanghui Wu, Michaela J. Day, Muriel T. Mafura, Javier Nunez-Garcia, Jackie J. Fenner, Meenaxi Sharma, Alieda van Essen-Zandbergen, Irene Rodríguez, Cindy Dierikx, Kristina Kadlec, Anne-Kathrin Schink, John Wain, Reiner Helmuth, Beatriz Guerra, Stefan Schwarz, John Threlfall, Martin J. Woodward, Neil Woodford, Nick Coldham, Dik Mevius

**Affiliations:** 1 Animal Health and Veterinary Laboratories Agency (AHVLA, Weybridge), Addlestone, United Kingdom; 2 Public Health England, London, United Kingdom; 3 Department of Bacteriology and TSEs, Central Veterinary Institute (CVI) of Wageningen, Lelystad, the Netherlands; 4 Department of Biological Safety, Federal Institute for Risk Assessment (BfR), Berlin, Germany; 5 Institute of Farm Animal Genetics, Friedrich-Loeffler-Institut (FLI), Neustadt-Mariensee, Germany; 6 Norwich Medical School, University of East Anglia, Norwich, United Kingdom; Institut National de la Recherche Agronomique, France

## Abstract

The putative virulence and antimicrobial resistance gene contents of extended spectrum β-lactamase (ESBL)-positive *E. coli* (n=629) isolated between 2005 and 2009 from humans, animals and animal food products in Germany, The Netherlands and the UK were compared using a microarray approach to test the suitability of this approach with regard to determining their similarities. A selection of isolates (n=313) were also analysed by multilocus sequence typing (MLST). Isolates harbouring *bla*_CTX-M-group-1_ dominated (66%, n=418) and originated from both animals and cases of human infections in all three countries; 23% (n=144) of all isolates contained both *bla*_CTX-M-group-1_ and *bla*_OXA-1-like_ genes, predominantly from humans (n=127) and UK cattle (n=15). The antimicrobial resistance and virulence gene profiles of this collection of isolates were highly diverse. A substantial number of human isolates (32%, n=87) did not share more than 40% similarity (based on the Jaccard coefficient) with animal isolates. A further 43% of human isolates from the three countries (n=117) were at least 40% similar to each other and to five isolates from UK cattle and one each from Dutch chicken meat and a German dog; the members of this group usually harboured genes such as *mph*(A), *mrx, aac*(6’)-*Ib*, *catB3*, *bla*_OXA-1-like_ and *bla*_CTX-M-group-1._ forty-four per cent of the MLST-typed isolates in this group belonged to ST131 (n=18) and 22% to ST405 (n=9), all from humans. Among animal isolates subjected to MLST (n=258), only 1.2% (n=3) were more than 70% similar to human isolates in gene profiles and shared the same MLST clonal complex with the corresponding human isolates. The results suggest that minimising human-to-human transmission is essential to control the spread of ESBL-positive *E. coli* in humans.

## Introduction

*Escherichia coli* is a commensal organism in people and animals but is also a causative agent of diarrhoea and extra-intestinal infections. It is responsible for an estimated 120 million cases of community-acquired urinary tract infections (UTI) diagnosed worldwide annually. It can also cause neonatal meningitis, pneumonia and surgical site infections. The sepsis-associated mortalities due to *E. coli* are estimated at 868,000 per year globally [1]. In England, Wales and Northern Ireland, *E. coli* has been the most common cause of bacteraemia for most years since 1990, with year-on-year increases to 27,055 reports in 2010 [2].

Since circa 2003, there has been a rapid and global increase in the occurrence of *E. coli* with resistance to oxyimino-cephalosporins due to the production of extended-spectrum β-lactamases (ESBLs). These isolates have emerged in both community and healthcare settings, are often also resistant to other antimicrobial agents, including fluoroquinolones, aminoglycosides and sulphonamides and resistant isolates have been associated with treatment failures [3]. Bacteraemia caused by ESBL producers can be associated with increased mortality, primarily because multi-resistance undermines the efficacy of empiric therapies, which are prescribed before the antimicrobial susceptibility of the infecting organism is known [4]. The CTX-M types of β-lactamases are the dominant family of ESBLs in *E. coli*, with particular subtypes associated with different geographic regions. However, the CTX-M-15 ESBL is pandemic and is often disseminated with the O25:H4-ST131 *E. coli* clone [5-7].

The occurrence of ESBL-positive *E. coli* in animals is also showing a general tendency to increase in some countries, among those bacteria isolated from the poultry gut as well as among those that contaminate food products [8-13]. The rise of community-acquired urinary tract infections caused by *E. coli* resistant to 3^rd^ - or 4^th^ - generation cephalosporins has been linked to international travel of people to countries of high prevalence [13-17] and to reservoirs of resistant bacteria in food-producing animals, especially poultry [13,18]. To explore the genetic relatedness of ESBL- and/or plasmid-mediated (p) AmpC-β-lactamase-producing *E. coli*, we used virulence and resistance gene microarrays as a convenient and rapid tool to investigate isolates from Germany, The Netherlands and the UK obtained from humans, food producing animals and animal food products. A subset of these isolates was also characterised by multi-locus sequence typing (MLST) to assist in elucidating the clonal relationship of isolates.

## Materials and Methods

### Sources of isolates

This study sought to compare ESBL/pAmpC-producing *E. coli* from the animal gut flora, animal-derived food products and from cases of human infections, especially from urinary tract infections (UTI) where the ESBL-producing isolates were often identified. For this, *E. coli* isolates that showed resistance to both ampicillin and cefotaxime, based on EUCAST criteria (www.eucast.org), and that had been isolated between 2005 and 2009 were included in this study. The isolates were from existing strain collections of the UK (AHVLA and Public Health England, formerly the Health Protection Agency), Germany (FLI and BfR) and The Netherlands (CVI) and had been obtained as part of national antimicrobial resistance surveillance programmes or from participants’ routine diagnostic or reference laboratory activities. The UK poultry isolates were from a structured survey [19] and those from cattle were derived from scanning surveillance of clinical diagnostic submissions to the 14 AHVLA regional laboratories across England and Wales [20]. The German isolates were from the collection at the National Reference Laboratory for *E. coli* (NRL-E*. coli*) and the NRL for Antimicrobial Resistance (NRL-AR) of the BfR (for food and animal isolates) and the National Reference Centre for Salmonella and other enterics (NRZ, human isolates, Robert Koch Institute). In addition, all putative ESBL-producing *E. coli* isolates from the BfT-GermVet collection were included [21]. The Dutch human isolates were selected from a national ESBL-prevalence study conducted in Public Health Laboratory Services in 2009 [13] and the animal isolates were selected from the collection of the NRL-AR (CVI). No field samples were specifically collected for this study. *E. coli* isolates (n= 629) from animals (n=295), animal-derived foods (n=59), humans (n=274) and an unknown source (n=1), originating from Germany (n=84), The Netherlands (n =254) and the UK (n =291) were analysed using microarrays (Table 1). MLST analysis was performed on 313 isolates isolated from different countries and host species.

**Table 1:** Distribution of selected β-lactamase genes in *E. coli* from different hosts^1^ and countries.

Host	Country	Percentage (no. of positive isolates)						
		Isolates (n)	*bla*_CTX-M-group-1_	*bla*_CTX-M-group-9_	*bla*_SHV_	*bla*_CMY-611_^2^	*bla*_CMY_11_^3^	*bla*_MOX-cmy_9_^4^
	All	274	74% (204)	8% (23)	11% (31)	0.7% (2)	3% (8)	7% (18)
	UK	152	74% (112)	6% (9)	16% (25)	0	5% (7)	10% (15)
Human	Germany	14	93% (13)	7% (1)	0	7% (1)	7% (1)	7% (1)
	Netherlands	108	73% (79)	12% (13)	6% (6)	1% (1)	0	2% (2)
	All	157	55% (86)	1% (1)	5% (8)	22% (35)	22% (34)	3% (5)
	UK	10	100%(10)	0	0	0	0	0
Chicken	Germany	11	18% (2)	0	0	72% (8)	73% (8)	0
	Netherlands	136	54% (74)	1% (1)	6% (8)	20% (27)	19% (26)	4% (5)
	All	133	63% (84)	31% (41)	1% (1)	3% (4)	2% (3)	1% (1)
	UK	95	57% (54)	43% (41)	0	2% (2)	2% (2)	0
Cattle	Germany	32	81% (26)	0	0	0	0	3% (1)
	Netherlands	6	67% (4)	0	17% (1)	33% (2)	17% (1)	0
	All	35	63% (22)	46% (16)				3% (1)
Turkey	UK	32	63% (20)	50% (16)	0	0	0	3% (1)
	Germany	2	50% (1)	0	0	0	0	0
	Netherlands	1	100% (1)	0	0	0	0	0
	All	17	94% (16)					
	UK	1	100% (1)	0	0	0	0	0
Pig	Germany	13	92% (12)	0	0	0	0	0
	Netherlands	3	100% (3)	0	0	0	0	0
All^5^		629	66% (418)	13% (81)	6% (40)	7% (44)	7% (48)	4% (25)

^1^ The precise sources of strains can be found in Table S1. Those from meat products were counted as if they were from the corresponding animals.

^2^ blaCMY-611, probe name and the sequence was found in blaCMY-2 (AB212086.1[93:118])and blaCMY-13 (AY339625.2[4669:4694:r])

^3^ blaCMY_11, probe name and the sequence was found in blaCMY-2 (AB212086.1[569:592])and blaCMY-13 (AY339625.2[4195:4218:r]

^4^ bla MOX-cmy_9, probe name and the sequence was in bla MOX-cmy_9 (AF357599.1[379:402])

^5^ There were also 6 isolates labelled as from poultry and 4 from cats/dogs from Germany, one UK isolate from sheep and 2 from unknown German animals.

### Microarray analysis

The principle and methodology of the microarray have been described previously [22-24] and the probe content was described by Geue et al. [25] and can be found at: http://alere-technologies.com/fileadmin/Media/Paper/Ecoli/Supplement_Geue__layout_E_coli.xlsx. In addition, a description of the probes that generated positive signals among isolates can be found in Table S1. The *bla*_CTX-M_ family probes identified genes only to group level (i.e. groups 1, 2, 8/25, 9) [26], and the *bla*_SHV_ and *bla*_TEM_ probes did not identify their precise alleles. The virulence probes allowed the detection of major pathotypes of *E. coli* such as enteropathogenic *E. coli* (EPEC), verotoxigenic *E. coli* (VTEC), enterotoxigenic (ETEC), extra-intestinal pathogenic *E. coli* (ExPEC) from humans and animals including avian pathogenic *E. coli* (APEC), and uropathogenic *E. coli* (UPEC) as described previously [23]. All probes were present on the array in duplicate, except probes for *sul3*, *tet*(A-G)*, bla*_TEM,_ which were singletons.

Hybridisation signals were normalised to the 50^th^ percentile of the signal intensities of control genes: *ihfA*, *gad*, *gapA*, *hemL* and *dnaE*. Normalised signal intensities with values >0.1 were in 95% in agreement with positive PCR results for *bla*_CTX-M-group-1_ (n=248) and *bla*_CTX-M-group-9_ (n=95) and 100% agreement with four *bla*_CTX-M-group-2_, *bla*_TEM_ (n=11) and *bla*_SHV_ (n=5) [27]; a gene was therefore considered present if the complementary array probe(s) produced normalised signal intensity that was >0.1. Cluster analysis of isolates was performed with Jaccard coefficients and Unweighted Pair Group Method with Arithmetic Mean (UPGMA, Bionumerics 5.1, Gent, Belgium). Isolates were grouped where stated to form larger clusters at different similarity levels; when the similarity level is not indicated, a 40% similarity threshold was used.

### Analysis of the diversity of isolates

To assess the diversity of isolates from each country and host species, different similarity cut-off points were used to group isolates into larger clusters to facilitate their comparison. The number of isolates within each cluster from each host species and country was counted and the Simpson’s Index of Diversity (1 -D) was calculated [28]. The distribution of isolates from each country and species among those clusters was analysed. Where possible, genes specifically associated with, and hence potentially indicative of major clusters, were identified.

### *E. coli* MLST typing

In order to estimate the relative importance of clonal spread versus horizontal gene transfer in the dissemination of ESBL genes, and to assess the association between virulence and resistance genes with genetic backbones of the host bacteria, isolates (n=313) representing different countries of origin or different host species were analysed by MLST according to the scheme described by Wirth et al. [29] following the guidelines given at http://mlst.ucc.ie/mlst/dbs/Ecoli

## Results

### Pathotypes of *E. coli* isolates and the presence of major ESBL/ pAmpC β-lactamase genes.

Virulence genes and antimicrobial resistance genes identified by microarrays are listed in Table S1. Only 0.5% of isolates (n=3) were ETEC and harboured the *fim41* (encoding F41 fimbria) and *sta1* (encoding a heat-stable toxin) genes [30]. Fewer than 7.5% of the isolates (n=46) were EPEC (that contained *eae* encoding intimin) or VTEC, harbouring both *eae* and *stx* genes (the latter gene encoding Shiga toxins).

The majority of isolates were either commensal or ExPEC and typically harbouring *prfB* (encoding P-related fimbria, n=241, 38% of the isolates) and/or *iroN* (encoding the enterobactin siderophore receptor protein) or microcin genes; 13% of isolates (n=80) contained *tsh* (encoding a temperature-sensitive haemagglutinin), a gene often associated with APEC [31]. Hybrid pathotypes of ExPEC and EPEC were also identified: three isolates (0.5%) harboured both *prfB* and *eae*. In addition, about 20% of Dutch chicken isolates (n=28) harboured EPEC-associated genes [30] and nearly half (n=13) also carried *tsh* (Table S1).

As expected, *bla*_CTX-M-group-1_ predominated amongst the CTX-M genes and was detected in 418 of 629 (66%) isolates (Table 1). Additionally, *bla*_OXA-1-like_ genes were found in combination with *bla*_CTX-M-group-1_ genes in about 23% of the isolates (n=144), mainly from humans (88%, n=127) and UK cattle (10%, n=15), but also in single isolates from Dutch chicken meat (ESBL428) and from a German pig (ESBL 154). While *bla*_CTX-M-group-1_ genes were widespread in all host species and in all three countries, *bla*_CTX-M-group-9_ genes were more common among cattle and turkey isolates from the UK (Table 1). Forty isolates were positive for *bla*_SHV,_ among which 6 and 25 were from Dutch and UK humans respectively, one and 8 from Dutch cattle and chickens respectively. More than half of the isolates (n=399) were *bla*_TEM_ positive, which were from all species and countries studied here. However, the array does not distinguish the ESBL and non-ESBL forms of *bla*_SHV_ and *bla*_TEM_ genes.

### Analysis of the diversity among the isolates

**Figure 1:**
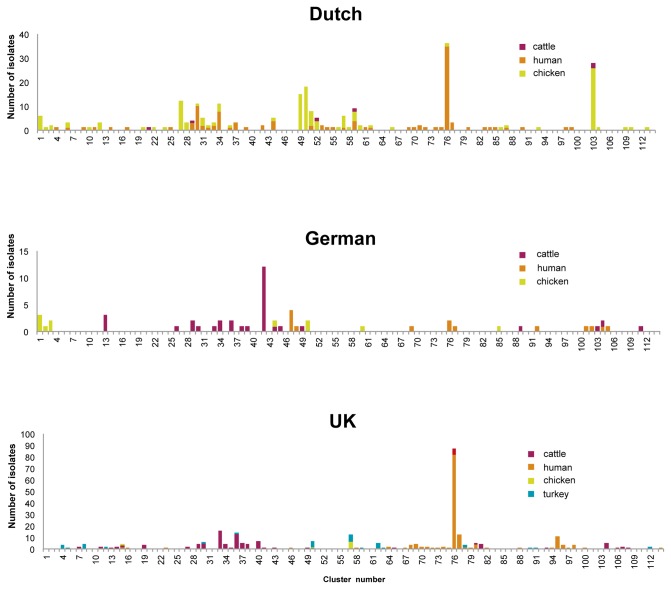
Isolates were grouped into 114 clusters at the 40% similarity level.

Isolates were clustered initially using the Jaccard coefficient and UPGMA (Bionumerics 5.1) based on the presence or absence of virulence and antimicrobial resistance genes. To identify isolates that may be related, similar isolates were grouped together into larger clusters. Isolates were divided into 12 clusters at 15% similarity and four of those clusters contained a single isolate, but approximately 76% of the 629 isolates were grouped in a single cluster (not shown). At 25% similarity, isolates were divided into 33 clusters of which 9 contained a single isolate, but 24%, 17% and 26% of the isolates were in three clusters respectively (data not shown). At 40% similarity, isolates were divided into 114 clusters (Figure 1). When raising the similarity threshold, the number of defined clusters increased further so that at 90% similarity, isolates were separated into 556 clusters, with 484 (87%) of them being singletons and only 8 clusters including isolates from different species.

**Figure 2:**
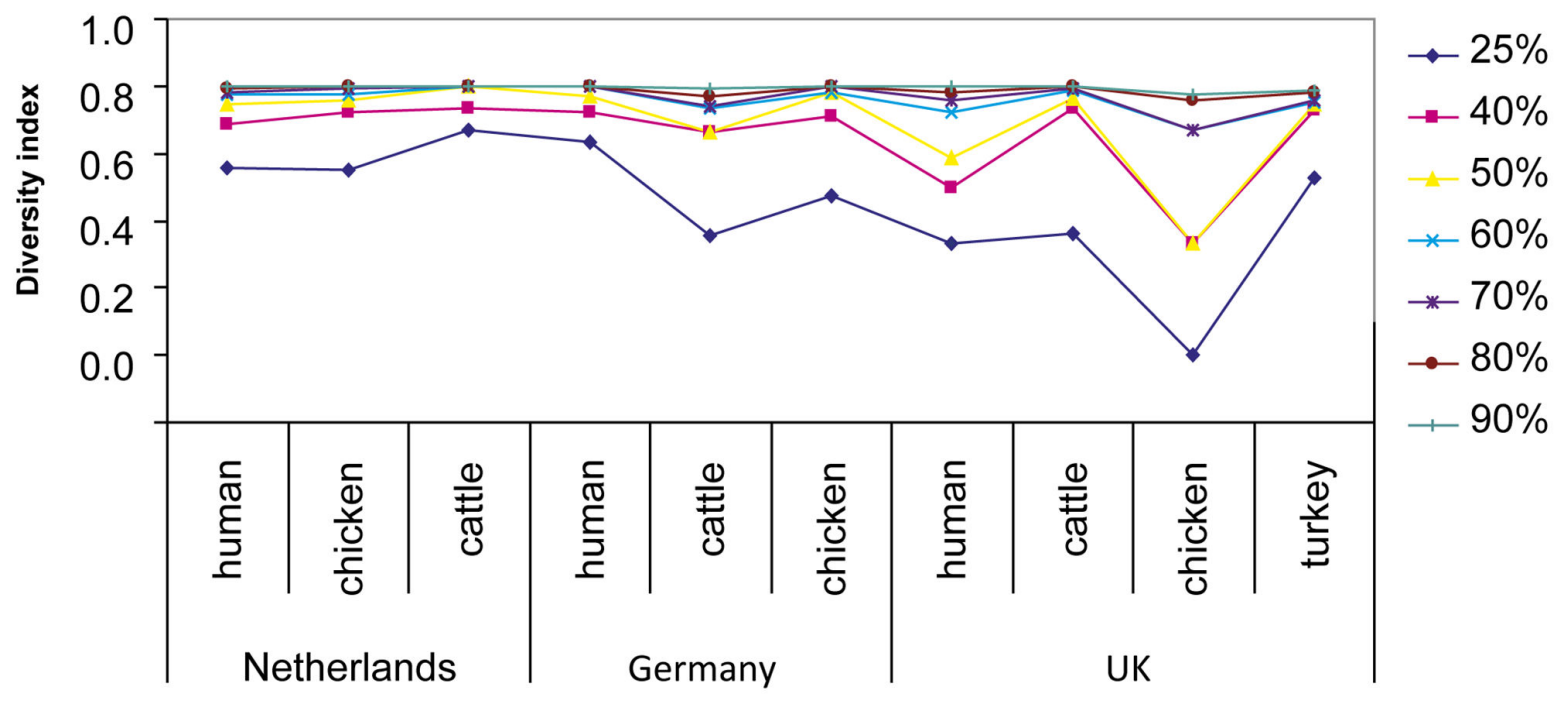
Simpson’s Index of Diversity (1-D) of isolates from different categories (country and host species) at the similarity levels from 25% to 90%.

To calculate the diversity of isolates, a series of cut-off points from 25% to 90% were used and the number of isolates from each category (country and species) in each cluster was counted and used to calculate the index of diversity and to plot the results (Figure 2). This approach showed that isolates from The Netherlands and Germany were very diverse and the differences were most pronounced between the 25% and 50% similarity levels. Only 14 human isolates from Germany were included; their high diversity was probably due to the inclusion of EPECs, whereas the human isolates from The Netherlands and the UK originated exclusively from urinary tract infections. With the exception of turkey isolates, the UK isolates showed the lowest overall diversity (Figure 2). As only three turkey isolates from Germany and The Netherlands were included in this study, a valid comparison with those from the UK could not be made.

### Comparison of isolates from different host species and countries

**Figure 3:**
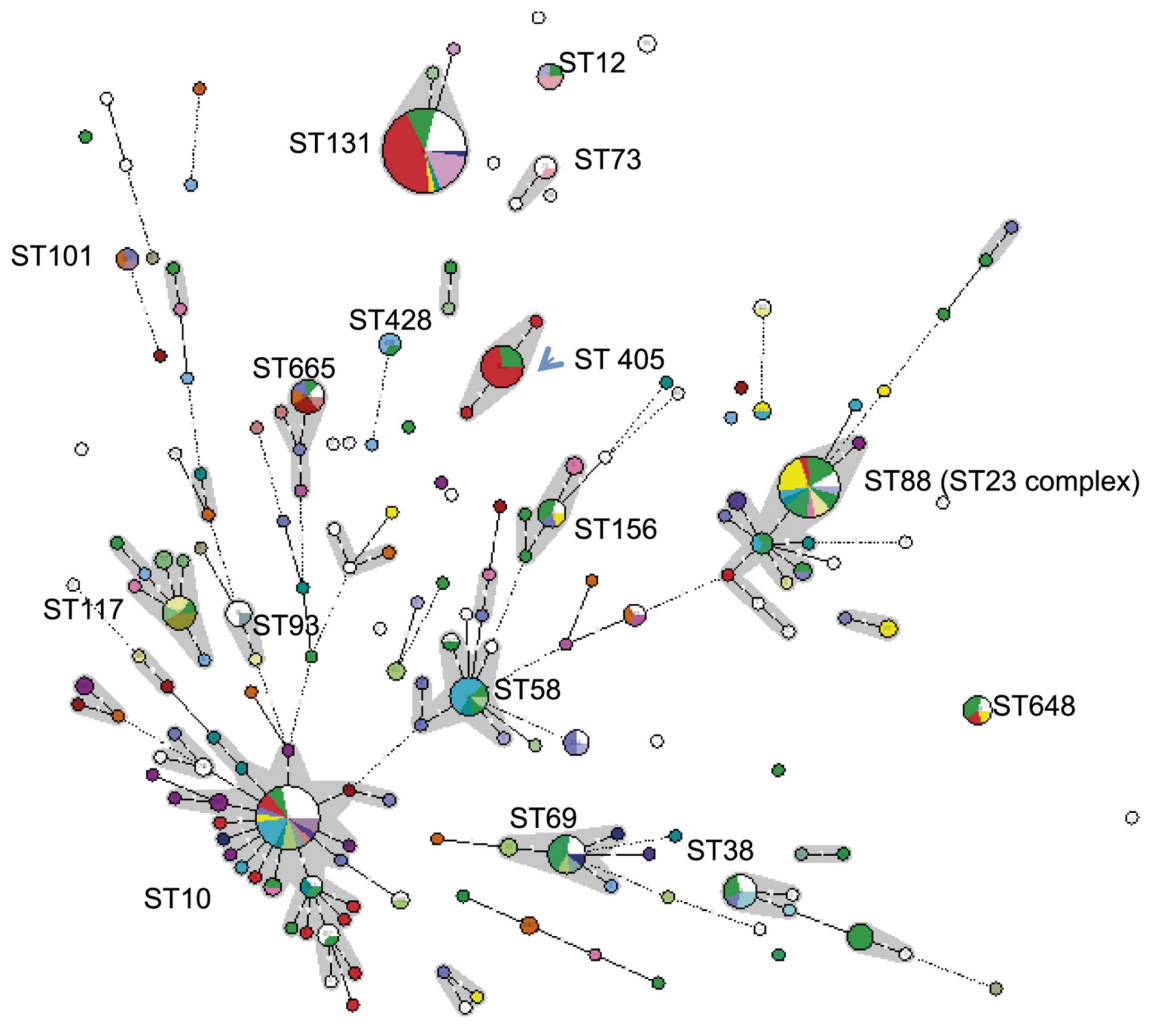
Minimal spanning tree constructed based on the MLST profiles of the 313 isolates (Bionumerics V6) and coloured according to the cluster numbers: red, cluster 76; purple, cluster 103; light blue, cluster 30; dark green, cluster 42; green, no array data and white, a mixture of rare cluster number.

While microarrays detect virulence and antimicrobial resistance genes that are often acquired through horizontal gene transfer, MLST provides information on the genetic backbone of host strains. In this study, MLST analysis was performed on 313 isolates in order to seek relationships between array clustering and multilocus sequence types (ST, Figure 3). The results showed that the major array clusters (e.g. 27, 30, 34, 42, 49, 50, 76, 103) all contained isolates of numerous STs, and that isolates of major STs (ST131 n=45, ST10 n=25, ST88 n=24, ST405 n=11, ST69 n=10, ST58 n=10) were divided among several very different array clusters (Figure 3). The largest array cluster, 76, consisted of 124 isolates of which 41 were typed by MLST [humans (n=36), UK cattle (n=3), Dutch chicken meat (n=1) and German dog (n=1)]. Among them, 44% (n=18, all from humans) belonged to ST131, 22% (n=9, all from humans) to ST405, 12% (n=5) to the ST10 complex and 5% (n=2) to the ST23 complex. Conversely, of 44 ST131 isolates only 43% were grouped in cluster 76. In the following sections, isolates from each host species were analysed together.

At the 40% similarity level, human isolates (n=274) were scattered among 69 clusters, but 43% (n=117) were in cluster 76 (Figure 1), including those from the UK (82/152), The Netherlands (33/108) and Germany (2/14). The isolates in this cluster were defined as the “common human type” and usually contained genes *mph*(A), encoding a macrolide 2’-phosphotransferase; *mrx*, encoding a putative protein; *aac*(6’)-*Ib*, encoding an aminoglycoside 6'-N-acetyltransferase; *catB3*, encoding a chloramphenicol acetyltransferase as well as the *bla*_OXA-1-like_ and *bla*_CTX-M-group-1_ β-lactamase genes. A small number of the human isolates from the UK (13%, n=20) and The Netherlands (1.9%, n=2) were grouped in clusters 94 to 100 which harboured less commonly detected resistance genes such as *bla*_SHV_ and/or *bla*_CTX-M-group-2_. In addition, 33.6% of isolates from cases of human infection in the Netherlands were in clusters 29-34, 36, 37, 39, 42 and 44 (Figure 1). All in all, a total of 32% (n=87) of human isolates were in 40/69 clusters, which consisted of only human isolates, and a further 43% in cluster 76 in which 95% were human isolates. On the other hand, 19 human isolates (7% of the total human isolates) were at least 70%, 13 human isolates (4.7%) were at least 80%, and five human isolates (1.8%) were at least 90% similar to animal isolates in array profiles based on the Jaccard coefficient.

Chicken isolates (n=157) were found among 40 clusters at the 40% similarity level; 20% of the total were in 13 of these clusters, which consisted only of chicken isolates. Dutch chicken isolates harbouring *bla*_CTX-M-group-1_ often also carried *intI1* (integrase gene of class 1 integron), while those from the UK often carried *intI2* (integrase gene of class 2 integron). The Dutch chicken meat isolate ESBL428 (ST88) found in cluster 76 shared the ST23 clonal complex (which is the second largest clonal complex within the *E. coli* MLST database) with a Dutch human isolate ESBL7 (ST 90), however, the similarity in array profiles between these two isolates was just over 40%. In addition, 21% (n=29) of Dutch chicken isolates shared 13 array clusters with human isolates. Also, 19% (n=26) of Dutch chicken isolates were in cluster 103 together with one German and two Dutch cattle isolates (Figure 1). The isolates in this cluster harboured genes often found among EPEC, such as *eae* and genes encoding type III secretion system, and half of those isolates also carried *tsh*.

Cattle isolates (n=133) were found among 41 clusters at the 40% similarity level and 23% (n=31) of the isolates were in 16/41 clusters that consisted of only cattle isolates. About 61% (n=81) of cattle isolates (from all three countries) shared clusters with less common human types (mostly Dutch) and 5% (n=5) of the cattle isolates from the UK were found in cluster 76. Two of the isolates in this cluster (ESBL569, ST167 and ESBL528, ST1284, both were within the ST10 clonal complex, the largest clonal complex within the *E. coli* MLST database) were at least 70% or 80% similar in array profiles to human isolates (ESBL73, ST617 from The Netherlands; ESBL760, ST167 and ESBL691, a double locus variant of ST1284, both were from the UK) that also belonged to this ST clonal complex. The UK cattle isolate ESBL528 shared more than 90% similarity in array profiles with the Dutch human isolate ESBL106. In array cluster 30, ESBL517 from UK cattle and ESBL101 from a Dutch human patient were both ST58 and were at least 80% similar in their array profiles. In terms of the differences in genes they harboured, *bla*_CTX-M-group-9_ was found in cattle isolates from the UK, but not in those from The Netherlands or Germany (Table 1). Among the six cattle isolates from The Netherlands, diverse genes including ESBL/AmpC genes *bla*_CMY_, *bla*_SHV_, and virulence genes *f17A, f17G* and *cif*, were detected. Cattle isolates from Germany (in cluster 42) harboured different genes as compared to cattle isolates from the UK (Figure 1).

Turkey isolates (n=35) were found in 16 clusters and 25.7% (n=9) of them were in five clusters which consisted of only turkey isolates, while 54% (from UK, n=17; Germany, n=1 and The Netherlands, n=1) of those shared clusters with human isolates from The Netherlands and the UK. All these turkey isolates were different from the “common human type”. Six turkey isolates were more than 70% similar and four were more than 80% similar to human isolates. One UK turkey isolate (ESBL 626) was 90% similar to the Dutch human isolate ESBL 80.

The 17 pig isolates, 13 of which were from Germany, were found in seven clusters. No cluster was observed that contained only pig isolates. All but one pig isolate (94%) clustered with human isolates. The majority (71%, n=12) harboured genes *mph*(A) and *mrx*; both genes were typically found among the “common human type”. Two pig isolates shared more than 70% similarity with human isolates, but their MLST profiles (single locus variant of ST348) were dissimilar to the respective human isolates ESBL116 (ST453) and ESBL80 (a double locus variant of ST167).

### Genes that were unevenly distributed among different host species and countries

Genes found significantly more or less common among isolates from humans and cattle in comparison with other species are summarized in Table 2. Antimicrobial resistance genes *bla*_OXA-1-like_, *aac*(*6*’)*-Ib*, *catB3* and virulence genes *iha* (encoding an adhesion)*, sat* (encoding serine protease precursor)*, prfB* were frequently found among human isolates, especially among the UK human isolates from urinary tract infections (Table S2). The long polar fimbria gene (*lpfA*) was less commonly represented among human isolates, especially among those from the UK (3.3%) as compared with those from The Netherlands (14.8%). The gene *floR*, which confers resistance to florfenicol, was seen among 33% of the cattle isolates, but was rare among isolates of other species (Table 2). Florfenicol is a fluorinated derivative of chloramphenicol approved for use in cattle in Europe since 1995 [32]. The *espP* gene that encodes serine protease autotransporter and *mcmA* that encodes for microcin M protein were significantly more common among cattle isolates than other species (Table 2). Genes that were more or less common in *E. coli* isolates from each country are listed in Tables S2 and S3.

**Table 2:** Genes that show significant differential presence among isolates from different species.

**Genes**	The proportion of isolates where the gene is present (The binomial 95% exact confidence interval)				
**More common among human Isolates**	Human	Chicken	Pig	Turkey	Cattle
*aac6*	0.482 (0.42,0.54)	0.006 (0,0.03)	0 (0,0.19)	0.057 (0,0.19)	0.075 (0.03,0.13)
*aac(6’)-Ib*	0.507 (0.44,0.56)	0.006 (0,0.03)	0 (0,0.19)	0.086 (0.01,0.23)	0.083 (0.04,0.14)
*bla*_OXA-1-like_	0.485 (0.42,0.54)	0.006 (0,0.03)	0.059 (0,0.28)	0 (0,0.1)	0.165 (0.1,0.24)
*catB3*	0.456 (0.39,0.51)	0.006 (0,0.03)	0 (0,0.19)	0 (0,0.1)	0.06 (0.02,0.11)
*iha*	0.504 (0.44,0.56)	0.019 (0,0.05)	0 (0,0.19)	0.086 (0.02,0.23)	0.211 (0.14,0.29)
*sat*	0.482 (0.42,0.54)	0 (0,0.02)	0 (0,0.19)	0.057 (0,0.19)	0.008 (0,0.04)
*prfB*	0.613 (0.55,0.67)	0.076 (0.04.0.13)	0.176 (0.03,0.43)	0.029 (0,0.14)	0.421 (0.33,0.51)
**Less common among human Isolates**					
*lpfA*	0.084 (0.05,0.12)	0.268 (0.2,0.34)	0.353 (0.14,0.61)	0.286 (0.14,0.46)	0.256 (0.18,0.33)
**More common among bovine Isolates**					
*floR*	0.026 (0.01,0.05)	0.057 (0.02,0.1)	0 (0,0.19)	0 (0,0.1)	0.331 (0.25,0.41)
*espP*	0.011 (0,0.03)	0.013 (0,0.04)	0.059 (0,0.28)	0.029 (0,0.14)	0.489 (0.4,0.57)
*mcmA*	0.113 (0.07,0.15)	0.019 (0,0.05)	0.059 (0,0.28)	0.057 (0,0.19)	0.444 (0.35,0.53)

## Discussion

In this study, we assessed the use a microarray approach to study gene relatedness among ESBL carrying *E. coli*. Many of the genes represented on the array are readily transmissible being encoded on mobile genetic elements. Therefore, we might anticipate greater diversity using this approach than other standard methods such as PFGE and MLST that focus on the genetic backbone of a strain. One advantage of the array approach that in this study detected genes encoding important phenotypes, in this case resistance and virulence, is the ability to identify known and novel (potentially emerging) gene associations.

A considerable number of human *E. coli* isolates (32%) did not share array clusters with animal isolates and a further 43% were concentrated in one cluster (cluster 76), which consisted mainly of human isolates. These results are consistent with a recent observation that the *bla*_CTX-M_-carrying *E. coli* isolates from UK chicken and turkey were different from the human-associated ST131 CTX-M-15 type in the UK [19]. In another study, 11% of human isolates were found to contain poultry-associated ESBL genes, plasmids and MLST types in The Netherlands [13], however, this study did not investigate virulence and additional antimicrobial resistance genes. In this study, only 1.2% (3/258) of characterized *E. coli* from animals were similar (as judged by gene content and clonal complex) to isolates from humans.

Nearly half of the human isolates investigated in this study were located in clusters surrounding cluster 76 and many of those belonged to ST131. Therefore, those isolates are likely to be similar to *E. coli* ST131-O25:H4-B2 that harboured *bla*_CTX-M-15_ plasmids (as *bla*_CTX-M-15_ belongs to *bla*_CTX-M-group-1_) [33,34]. Moreover, ST131 was found among 11 different array clusters, which is consistent with the reports that O25:H4-B2- ST131 isolates carry various *bla*_CTX-M_ types on a number of different plasmid types (Inc FII, FIA-FIB, FIA, FII-FIA, I1, N and Y for example) [35,36]. The O25:H4-ST131 clone has been found around the globe [5-7] and its spread between humans has been facilitated by international travel [37]. ST405 was identified as another important resistant sequence type among human isolates that shared similar virulence and antimicrobial resistance genes with many ST131 isolates.

As many human isolates from the three countries were highly similar to each other, the widespread human-to-human transmission of ESBL-producing *E. coli* is a strong possibility. Nevertheless the potential threat posed by animals or animal food products as sources for human ESBL-positive isolates cannot be ignored [13]. When clonally related CTX-M-producing *E. coli* were identified in community settings, common infection sources such as food or water were often suspected [38,39]. The dissemination of resistance genes in *E. coli* may occur through multiple routes. It is the opinion of EFSA that “transmission of ESBL genes, plasmids and clones from poultry to humans is most likely to occur through the food chain” [40], however the data presented in a recent review showed that considerable differences in ESBL types between poultry and humans in Europe exist, leaving the question open as to what extent livestock has contributed to the spread of ESBLs in humans [41]. The ESBL genes are often located on plasmids and are therefore likely to disseminate *via* horizontal gene transfer, as described recently for the transmission of a *bla*_CTX-M-14_-carrying plasmid [42]. This study did not seek to confirm routes of transmission between man and animals or animals and man, but to investigate whether organisms similar at the genetic level were present in different animal species and man, indicating possible epidemiological links. Such links might be consistent with food-borne transmission from animal to man, but equally, might indicate exposure of animals to human faecal bacteria in sewage or flooding incidents or by other routes.

This work demonstrated that microarray analysis is a useful tool for detecting the genetic diversity of a large number of ESBL-producing *E. coli* isolates although we recognize one weakness regarding the inability to differentiate group and sub-groups of ESBL in sufficient depth. The amount of data generated by this small array platform is more manageable compared with the whole genome glass slide microarrays [43,44] or whole genome sequencing. The whole genome arrays provide more information on genes located on the chromosome, but less information on virulence genes and mobile genetic elements, such as plasmids, where the ESBL and pAmpC-β-lactamase genes are often located. The associations of some genes with *E. coli* from certain animal species also suggest that a targeted approach can provide useful epidemiological information.

The isolates analysed in this study were highly diverse especially those from The Netherlands and Germany. The results do not necessarily reflect the diversity of isolates in that country and that species in general, because the isolates were collected from different surveillance programmes and between 2005 and 2009. Some of the observed diversity might therefore reflect differences in the sampling strategies used and in the time periods when samples were taken. There is also the need to be particularly cautious when only very small numbers of isolates were analysed (e.g. those from Germany) and indeed a recommendation for future work is the application of agreed standardised sampling protocols.

The clusters defined by the arrays showed poor congruence with MLST data, i.e. isolates of different, unrelated STs were found within the same array cluster, and isolates sharing the same ST were found in different array clusters; this observation is consistent with a previous study on ExPEC isolates from animals, where no correlation between virulence and antimicrobial resistance genes and genetic backbone of the host strain was found [45]. This was anticipated given the mobility and potential transience of many of the genes represented on the array.

In conclusion, the microarray analysis of ESBL- and AmpC- producing *E. coli* isolates showed a high diversity in virulence and antimicrobial resistance gene contents. The array profiles of the majority of isolates from humans were generally different from those isolated from animals, while many human isolates from the three countries were highly similar in both array profiles and MLST types. Thus, approaches to minimize human-to-human transmission are essential for controlling the spread of ESBL-positive *E. coli*. From public health perspective, ESBL-positive *E. coli* from animals may represent a reservoir of virulence and resistance genes rather than being the direct cause of infections in humans.

## Supporting Information

Table S1 **Microarray results of isolates used in this study.**
(XLSX)

Table S2 **Genes that show significant differential presence among human isolates from The Netherlands and the UK.**
(XLS)

Table S3 **Genes that show significant differential presence among isolates from different countries.**
(XLS)
