# Correction: Comparative Analysis of ESBL-Positive *Escherichia coli* Isolates from Animals and Humans from the UK, The Netherlands and Germany

**DOI:** 10.1371/journal.pone.0108834

**Published:** 2014-09-17

**Authors:** The PLOS ONE Staff

Dr. Marie Chattaway should be included as an author for this article. She should be listed as the 12th author, and her affiliation is: 2. Public Health England, London, United Kingdom. The contributions of this author are as follows: Performed the Experiments, analyzed the data.
